# The impact of artisanal gold mining, ore processing and mineralization on water quality in Marmato, Colombia

**DOI:** 10.1007/s10653-021-00898-y

**Published:** 2021-04-11

**Authors:** Keith W. Torrance, Stewart D. Redwood, Alessandro Cecchi

**Affiliations:** 1 University of Strathclyde, Glasgow, UK; 2 Consulting Geologist, Panama, Panama; 3 Gran Colombia Gold Corp., Medellín, Colombia

**Keywords:** Marmato, Water quality, Artisanal and small-scale gold mining, Colombia, Arsenic speciation, Mercury

## Abstract

Marmato, Colombia, has been an important centre of gold mining since before the first Spanish colonizers arrived in 1536. The Marmato deposit is hosted in a dacite and andesite porphyry stock as sheeted sulphide-rich veinlet systems. The district is currently experiencing a surge in both major mining projects and artisanal mining, driven by sustained high gold prices. Ore from small-scale and artisanal gold mining is processed in numerous small mills (*entables)* around Marmato, which impact surface water quality through the discharge of milled waste rock slurry, highly alkaline cyanide-treated effluent, and high dissolved metal loads. To investigate the impact of artisanal mining and ore processing, water samples were collected in January 2012 from streams around Marmato. The average dissolved metal concentrations in impacted streams were Zn, 78 mg L^−1^; Pb, 0.43 mg L^−1^; Cu, 403 µg L^−1^ Cd, 255 µg L^−1^; As, 235 µg L^−1^; Ni, 67 µg L^−1^; Co, 55 µg L^−1^; Sb, 7 µg L^−1^; and Hg, 42 ng L^−1^, exceeding World Health Organization drinking water guidelines. In addition, arsenic speciation was conducted in-situ and indicated that 91–95% of inorganic arsenic species is in the form of As(V). Spatial analysis of the data suggests that entables processing ore for artisanal miners are the main contributor to water pollution, with high sediment loads, alkalinity and elevated concentrations of dissolved arsenic, cadmium, mercury and lead, caused by the processing of gold-bearing sulphides in the entables. Geochemical data from surface water were compared to a comprehensive data set of whole rock analyses from drill core and channel samples from the deposit, indicating that the deposit is significantly enriched in gold, silver, lead, zinc, arsenic, antimony, and cadmium compared to crustal averages, which is reflected in the surface water geochemistry. However, elevated mercury levels in surface water cannot be explained by enrichment of mercury in the deposit and strongly suggest that mercury is being added to concentrates during ore processing to amalgamate fine gold.

## Introduction

Colombia has a long and rich history of gold mining, dating back to the pre-Colonial era (Brooks et al., 2016). Gold mining is an important economic driver in the impoverished rural regions in Colombia, with around 300,000 miners working in the artisanal and small-scale gold mining (ASGM) sector on a mainly subsistence basis, producing an estimated 54 metric tonnes (Mt) of gold annually (Cordy et al., 2011). Many of these mines operate illegally under Colombian law, without valid mining claims, property title or environmental permits. However, as these mines represent one of the few sources of employment in rural areas, they are generally tolerated by the authorities. The improved security situation in Colombia has made it an active exploration target for foreign mining companies.

It is known that water quality in the Marmato district is impacted by mining; Prieto analysed waters in the Marmato District (G. Prieto, 1998) and reported appreciable quantities of dissolved metals, including Zn, Cd, Cu, and As, with concentrations of dissolved solids up to 39,000 mg L^−1^. The purpose of this study was to document the geochemistry of surface water around Marmato, to determine the types of metal pollutants present, and to define their spatial relationship to artisanal mining and ore processing.

**Fig. 1:**
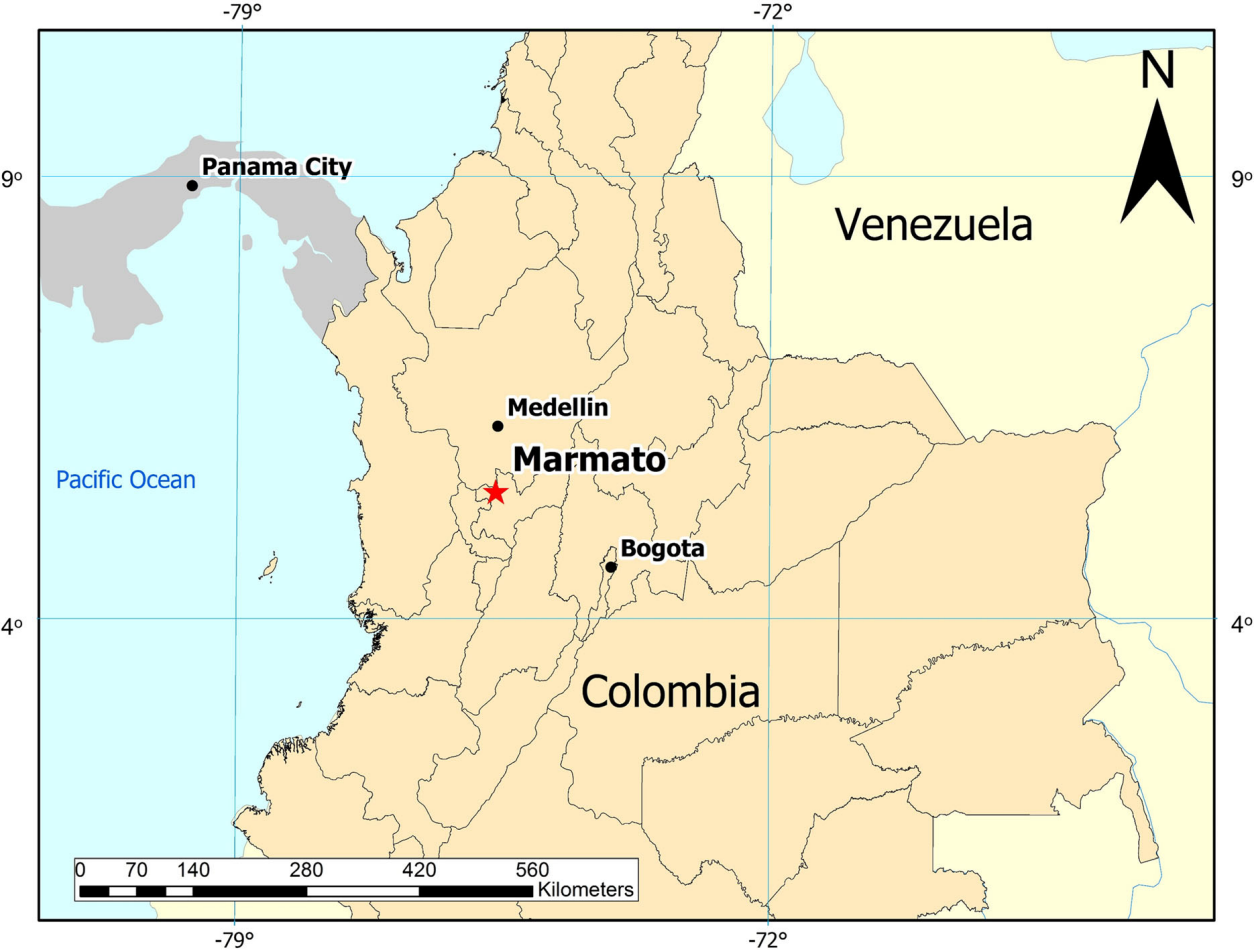
Large-scale map of Colombia showing the location of the Marmato mining district in Caldas Department, Colombia

The town of Marmato (5.47°N, 75.60°W) is in the Caldas Department of Colombia, about 80 km south of Medellin (Fig. 1). Marmato has been a centre of hard rock gold mining since its founding by the Spanish conquistadors in 1532, although gold in the area was exploited in pre-Colonial times by the Quimbaya people (Redwood, 2011). In 2012, approximately 2000 residents were directly employed in mining and ore processing out of a total municipal population of 10,000, with numerous small-scale mines and adits around the town and further up Marmato Mountain. There is one large-scale modern underground mine at an elevation of 1180 m, operated by Mineros Nacionales S.A., a subsidiary of Caldas Gold Corporation. An estimated 1.75–2.25 million ounces (Moz.) of gold was recovered from the Marmato district by 2011 (Redwood, 2011), but the district contains additional large, low-grade ore bodies that could be economically mined by bulk methods.

Marmato is situated at a mean elevation of 1,300 m on a steep hillside above the valley of the Rio Cauca, which flows northward towards the Caribbean. The district is readily accessible by road, being connected to the Pan-American Highway by a short length of mostly paved road. The terrain is exceptionally steep, rising over 600 m in 2 km, so landslides are a frequent problem after heavy rain, blocking roads and damaging property within the town. Marmato has an equatorial climate, described as moist tropical climate, ‘Am’ by the Köppen and Geiger classification (Peel et al., 2007). Temperatures are warm year-round, with maximum temperatures ranging from 28.7 to 31.6 °C, and minimum temperatures in the range of 17.4–18.7 °C (Knight Piésold Consulting, 2012). The average annual rainfall is 1889 mm per year, with drier periods in January, when sampling was carried out, and July (Knight Piésold Consulting, 2012). Wetter periods are in typically in spring and fall.

Marmato Mountain has been an exploration target of Gran Colombia Gold Corporation who originally defined open pit resources in the measured and indicated categories estimated at 11.8 Moz Au and 80.3 Moz Ag in 409.7 Mt of ore grading 0.90 g/t Au and 6.1 g/t Ag (Parsons & Armitage, 2012). Gran Colombia Gold originally proposed developing an open pit mine, but in 2017, their focus switched to evaluate underground bulk mining potential following discovery of the Marmato Deeps deposit. The current combined underground resource comprises 39.40 Mt at 3.20 g/t Au and 8.7 g/t Ag in the measured and indicated categories (4.09 Moz Au and 11.05 Moz Ag) plus 26.40 Mt at 2.60 g/t Au and 4.4 g/t Ag inferred (2.17 Moz Au and 3.73 Moz Ag) in veins, “underground porphyry” (veinlets) and Deeps deposits (Parsons et al., 2020).

**Fig. 2:**
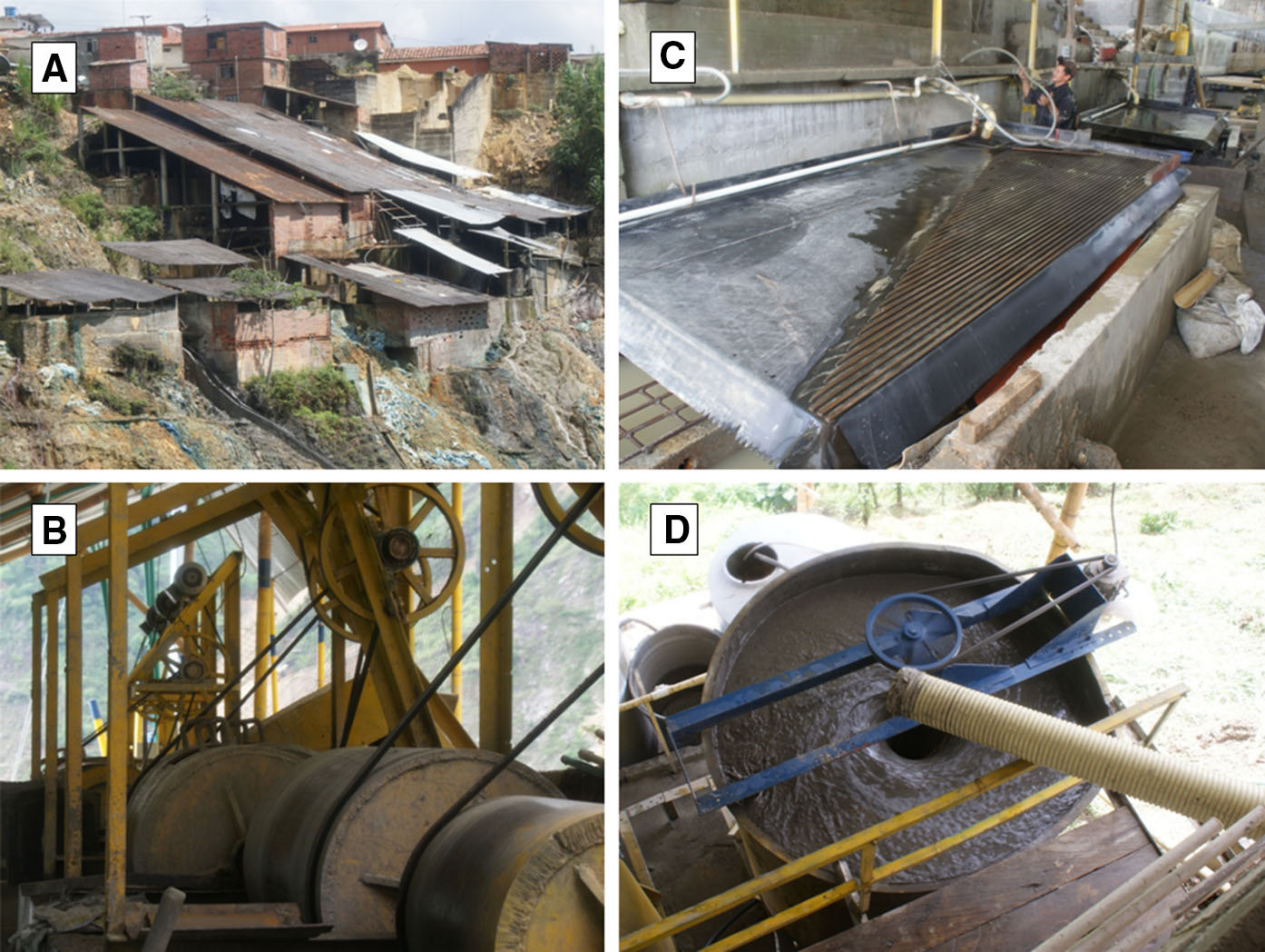
**a** Gold ore processing in an entable in Marmato. **b** Ore is first crushed by jaw crusher and then **(b)** ground in small ball mills called *cocos*. **c** Gold and auriferous pyrite are separated by gravity using a Wilfley table to concentrate the denser gold and pyrite grains. **d** Tailings are further concentrated in cyclones, then treated with cyanide to dissolve the remaining gold

ASGM and ore processing are the most visible sources of water pollution around Marmato. There several hundred artisanal mines around Marmato, exploiting gold-rich pyrite veins in the upper portion (Zona Alta) of Marmato Mountain. A typical small-scale mine consists of a single narrow adit tunnelled into the hillside to a length of 100 m or less, following a gold-rich pyrite vein. As most mines lack basic ventilation, these adits do not extend very deep into the hillside. The sulphide-rich ore is manually mined from adits and loaded into ore carts, which are pushed to the mine entrance for loading on to trucks or buckets on aerial cable-ways for transport to the ninety or so local processing mills, called *entables*, as shown in Fig. 2.

At the entables, the ore is crushed, then finely ground using small ball mills, known as *cocos*, to separate free gold and gold-bearing pyrite from the rock gangue. Denser gold flakes and sulphides are concentrated by gravimetry using cyclones and Wilfley tables (Fig. 2c). Gold is recovered from the concentrate by physical processes and by mercury amalgamation. Tailings from the enrichment processes are subsequently treated with sodium cyanide solution, which forms a complex with any remaining gold in the ore according to the Elsner equation (Eq. 1), allowing additional recovery.

1 $$4{\text{Au }} + \, 8{\text{NaCN }} + {\text{ O}}_{2} + \, 2{\text{H}}_{2} {\text{O}} \rightleftarrows 4{\text{Na}}\left[ {{\text{Au}}\left( {{\text{CN}}} \right)_{2} } \right] \, + 4{\text{NaOH}}$$

**Fig. 3:**
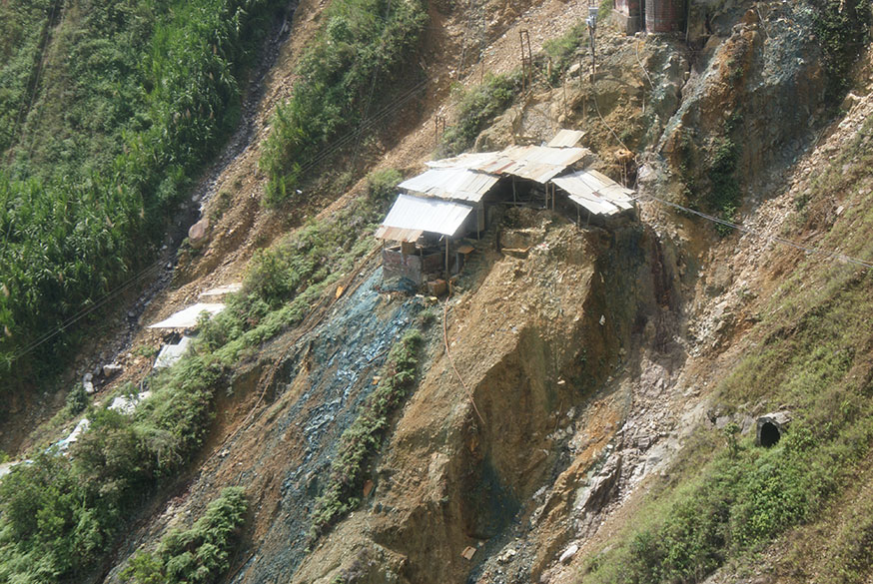
A small entable near Marmato, Colombia, built on a precarious slope to process gold ore. Note the distinctive blue ferrocyanide staining from cyanide discharges from the mill. A small adit is visible in the right foreground

The addition of sodium cyanide to the milled ore creates an effluent that is highly alkaline; sodium hydroxide is also added to maintain a high pH to favour cyanide complexation and suppress toxic hydrogen cyanide generation. Effluent from processing is discharged directly to drainage channels without basic treatment or settling ponds. This is the most visible source of pollution entering the streams that flow down the mountain into the Rio Cauca. Downgradient of each entable is a distinctive Prussian blue stain caused by the formation of ferrocyanide complexes as the waste effluent is discarded (Fig. 3).

**Fig. 4:**
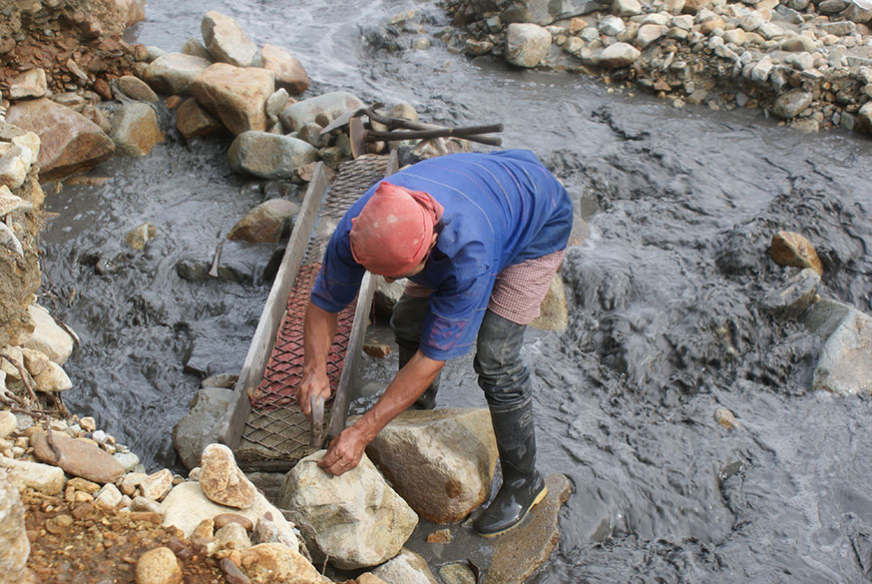
A barequero working a sluice box in Qda. Cascabel at its confluence with the Rio Cauca. Note the high quantities of sediment in the creek from slurry discharged from the entables

By-products from the milling and recovery process are discharged as a grey slurry (Fig. 4). A suspended sediment load of 40 g of sediment per liter was measured in one sample, downstream of the entables. In addition to the adit mines, small numbers of artisanal miners (*barequeros*) work the streams using pans, sluice boxes and riffle boards to recover any gold overlooked during processing (Fig. 4).

With an improving security situation, the Marmato district has attracted the attention of international mineral exploration companies looking to employ large-scale modern bulk mining methods with efficient gold recovery technologies. Gran Colombia Gold Corporation is exploring and developing the Marmato deposit and filed a NI 43–101 with the Toronto Stock Exchange in 2012 (Parsons & Armitage, 2012), which included an outline plan for a large open pit mine. This filing included preliminary environmental baseline studies. In October 2017, Gran Colombia Gold (Gran Colombia Gold Corp. press release, 4 October 2017) announced that they plan to develop the Marmato deposit as an expansion of their existing underground mine and released a preliminary economic assessment in 2019 (Parsons et al., 2019) and a pre-feasibility study in 2020 (Parsons et al., 2020), as well as spinning out the project into a new company, Caldas Gold Corporation. A consequence of this decision is that Marmato will not have to relocate as originally proposed and that artisanal gold mining within the previously planned open pit boundary will continue.

**Fig. 5:**
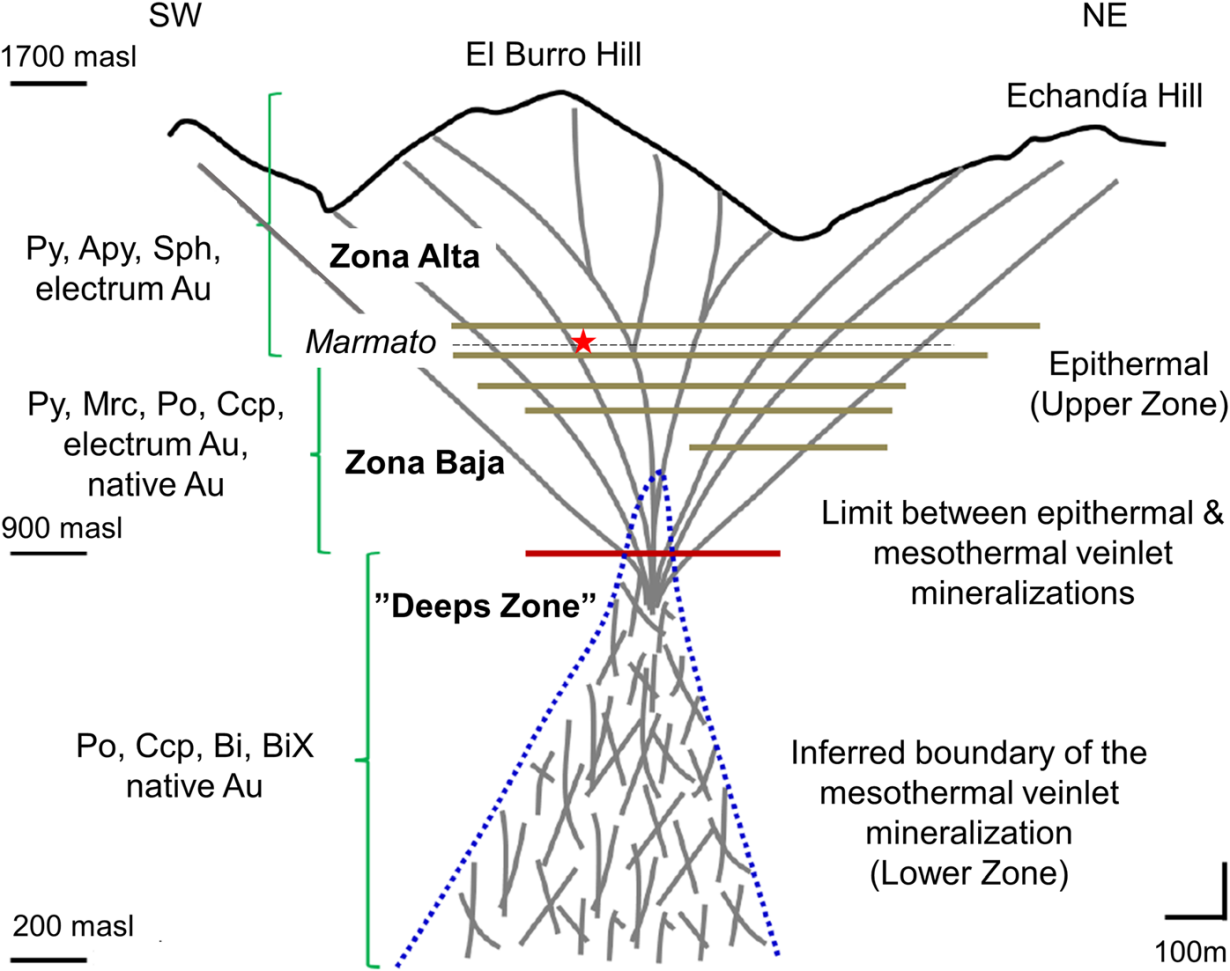
Cross section of the Marmato deposit. Adapted from (Santacruz et al., 2018), showing the different mining zone. Abbreviations: Py—pyrite; Ccp—chalcopyrite; Po—pyrrhotite; Apy—arsenopyrite; Sph—sphalerite (marmatite); Mrc—marcasite; Bi—bismuth; BiX—bismuth minerals; Au—gold

Marmato lies within the Romeral Terrane, an oceanic terrane of probable Late Jurassic to Early Cretaceous age that was accreted to the continental margin along the N-S trending Romeral Fault in the Aptian. This is partly covered by Neogene sediments and volcanic rocks, into which the composite Marmato stock was intruded. Gold mineralization is hosted by a composite andesite to dacite porphyry and is late stage, post-intrusion. Five porphyry pulses have been identified, named P1 to P5 from oldest to youngest. The age of the porphyry intrusions is bracketed by laser ablation ICP-MS ^206^Pb/^238^U zircon dates of the P1 dacite stock of 6.576 ± 0.075 million years (Ma) and P5 dacite dikes of 5.75 ± 0.11 Ma (Santacruz et al., ). The age of mineralization was dated by ^40^Ar/^39^Ar analyses of adularia in veins with plateau ages between 6.95 ± 0.02 Ma and 5.96 ± 0.02 Ma (Santacruz et al., 2018). Mineralization is structurally controlled with dominant NW and WNW trends, which developed from reactivated basement structures and as Riedel shears under WNW-ESE compression in a sinistral transpressional shear system. Mineralization extends over 1400 m vertically and is open at depth. Two main zones of mineralization have been identified. The Upper Zone (Fig. 5) between 1600 and 900 m above sea level (m.a.s.l.), mainly comprises massive, sulphide-rich, relatively quartz-poor, gold-bearing base metal veins and veinlets with sericite-illite–smectite-ankerite-pyrite wall-rock alteration which overprints pervasive propylitic alteration. This zone has a mineral assemblage of pyrite-arsenopyrite-Fe rich sphalerite (marmatite)-pyrrhotite-chalcopyrite and electrum (average 65% Au, 35% Ag). The Lower Zone, below 900 m.a.s.l. and is still open at depth below 200 m.a.s.l., comprises sulphide and quartz-rich veinlets and minor veins, with a mineral assemblage characterized by pyrrhotite-chalcopyrite-bismuth minerals and free gold (average 94% Au, 6% Ag).

## Materials and methods

### Surface water quality measurements

**Fig. 6:**
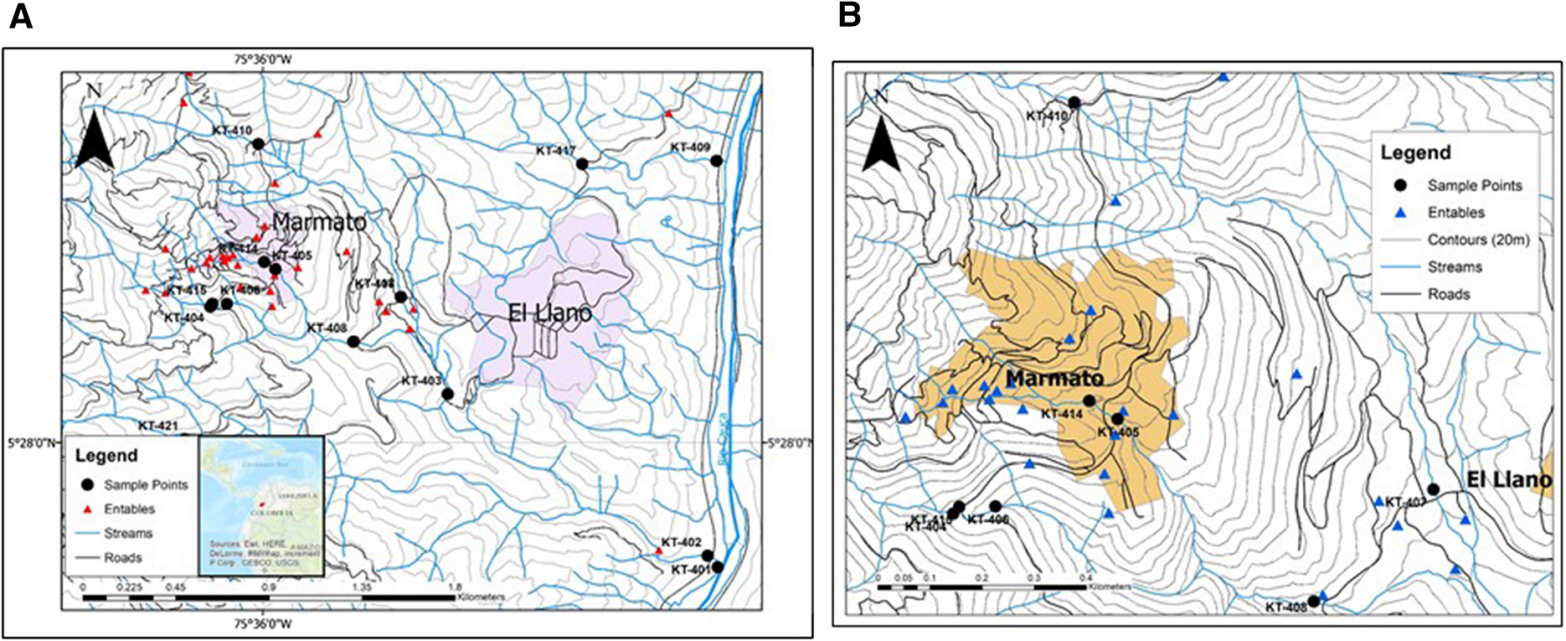
**a** Map of the Marmato district, Colombia showing the location of all sample collection points and entables. **b**. Sampling locations around the city of Marmato

For this study, twenty sample sites in streams were selected across the district based on their proximity to active mines and entables, as shown in Fig. 6. Streams originate as springs and seeps at higher elevations, flowing from west to east into the Rio Cauca, but are largely fed by precipitation with minimal baseflow during dry seasons (JBR Environmental Consultants Inc., 2011). Water samples were collected over two days in January 2012, and their locations were determined using a Garmin Model Oregon 450 global positioning unit.

Field data are presented in Appendix, Table 2. Surface water samples were collected at each sampling point (Fig. 6), following the protocols previously described (Torrance et al., 2012a). Water parameters, including temperature, pH, conductivity, and oxygen reduction potential (ORP), were measured at each location using a Hanna HI9828 multi-parameter meter. Alkalinity was measured in the field using a KI-9810 CHEMetrics Titrets^©^ titration kit.

### Arsenic speciation determination

The Marmato deposit has characteristics of an intermediate sulfidation epithermal system (Santacruz et al., 2014), which usually has a spatially wide arsenic signature. An additional goal of this investigation was to characterize arsenic speciation in surface water which may control its mobility. A simple field technique was used to separate arsenite species, [As(III)], and arsenate species, [As(V)], as first proposed and qualified by Ficklin (Ficklin, 1983), using a strong anion-exchange resin. An updated method (Wilkie & Hering, 1998) was developed with further modifications (Haque & Johannesson, 2006; Munk et al. 2011; Torrance et al., 2012a). Organic arsenic compounds may elute with As(III) and potentially skew the speciation (Miller, 2000), but is unlikely to be a concern with mineral-derived arsenic sources. Other authors (Issa et al., 2010) recommended pre-concentration of As(III) after separation in samples where As(V) dominates, but this was not found to be an issue at Marmato, where As(III) is always present above detectable limits.

In the field, a 50-mL sample for As speciation was filtered from the bulk sample into a pre-cleaned 50-mL HDPE tube, containing 100 µL of ultra-pure nitric acid. This was immediately passed through a Poly-Prep® anion-exchange column, prepared in 10 mL, 0.8 × 4 cm Poly-Prep® columns, purchased from Bio-Rad Laboratories (Hercules, CA). The columns were filled with an analytical grade anion-exchange resin Bio-Rad AG® 1-X8 (50–100 mesh, chloride form), which had been converted in bulk to the acetate form, replacing the Cl^−^ function group with an acetate group. Conversion of the 1-X8 resin from the chloride form to the acetate form was accomplished in the laboratory by washing 50 g resin with 150 mL of 1 M NaOH solution (J. T. Baker, Phillipsburg, NJ), rinsing with NANOpure™ water and repeating two times until the pH of the rinse was neutral. The resin was then washed with approximately 150 mL of 1 M acetic acid (BDH Aristar Ultra) a total of four times and rinsed with NANOpure™ water until neutral after each step. This quantity was sufficient to pack approximately twenty-five 0.8 × 4 cm Bio-Rad Poly-Prep® ion exchange columns, at 2 mL per column, which were stored at 4° C before use.

An aliquot, at a pH of less than 3, was passed through the column in increments of 5 mL until all the sub-sample had passed through and collected in a 60 mL HDPE bottle. As the sample passed through the column, oxy-anionic As(V) species, such as H_2_AsO^4−^, were exchanged with the acetate functional group in the resin, while neutral-charged As(III) species passed through the column. This allowed the quantities of both As species present in the sample to be determined by comparison of the total dissolved As analysis of both aliquots by ICP-MS. Samples were stored at 4 °C in the dark until analysis. Duplicate samples were taken to verify the quality of the data. A field blank (KT-419) was processed with the samples in the field and showed no elevated metals, except for Cu (~ 3.7 µg L^−1^).

Anions were measured using a Dionex® BioIC ion chromatography instrument. Total metal concentrations were determined by inductively coupled plasma mass spectrometry (ICP-MS), using an Agilent 7700× instrument. Total Hg was determined using a PS Analytical Millennium Merlin Atomic Fluorescence Spectrometer (AFS).

## Results and discussion

Concentrations of selected dissolved metals in surface water are presented in Appendix (Table 3). The average dissolved metal concentrations^1^ from streams impacted by ore processing discharges were: Zn, 78 mg L^−1^; Pb, 0.43 mg L^−1^; Cu, 403 µg L^−1^ Cd, 255 µg L^−1^; As, 235 µg L^−1^; Ni, 67 µg L^−1^; Co, 55 µg L^−1^; Sb, 7 µg L^−1^; and Hg, 42 ngL^−1^. For those metals that the World Health Organization (WHO) has established drinking water guidance (WHO, 2011), average dissolved Cd concentrations are 80 times the WHO guidance value of 3 µg L^−1^; average dissolved Pb concentrations are 43 times the WHO guidance value of 10 µg L^−1^, and average dissolved As concentrations are 23 times the WHO guidance value of 10 µg L^−1^. These represents a significant impairment of water quality rendering it unsuitable for drinking water and livestock watering.

### Arsenic

**Fig. 7:**
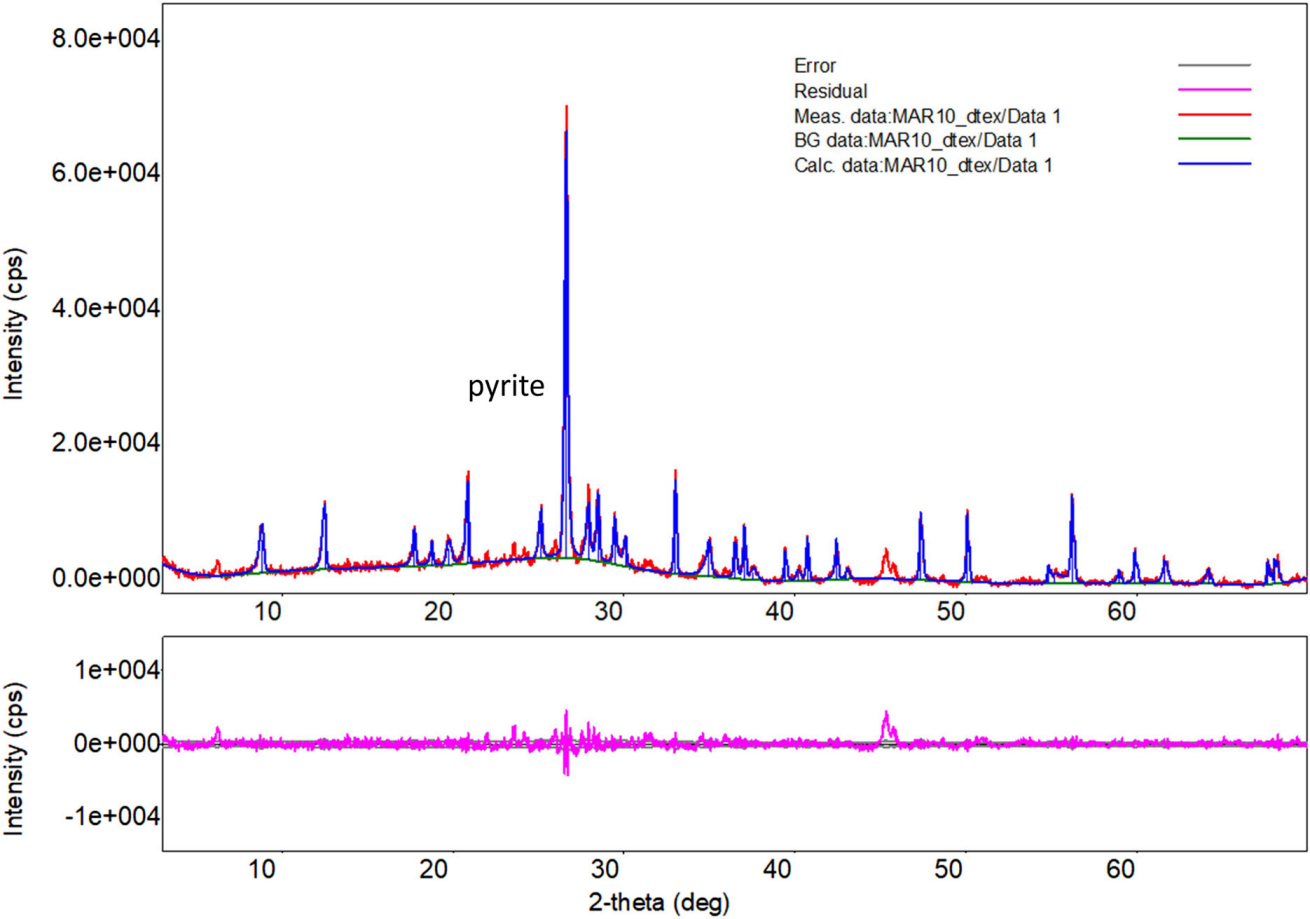
Powder XRD pattern of suspended sediment collected at KT-405, showing dominant peaks for pyrite

Latin America has well documented occurrences of arsenic exposure including previous studies from Marmato (Bundschuh et al., 2012). Total dissolved As in surface water ranged from 6 to 3521 µg L^−1^, with higher values in water that was visibly affected by run-off from ore processing. XRD analysis of solid particulates filtered from stream water indicates a high proportion of pyrite (Fig. 7), and it seems likely that As enters the watershed from the dissolution of pyrite and arsenopyrite during ore concentration. The upper zone of the deposit, Zona Alta, which is worked exclusively by small-scale miners, contains more arsenopyrite than the lower Zona Baja (Santacruz et al., 2018).

Dissolved arsenic concentrations in the unpolluted Quebrada (Qda.) Los Indios are also elevated at 90–140 µg L^−1^ which suggests that background As concentration is naturally elevated in the region. This is confirmed by whole rock assays determined during the exploration program, which had an average As concentration of 65 ppm.

**Fig. 8:**
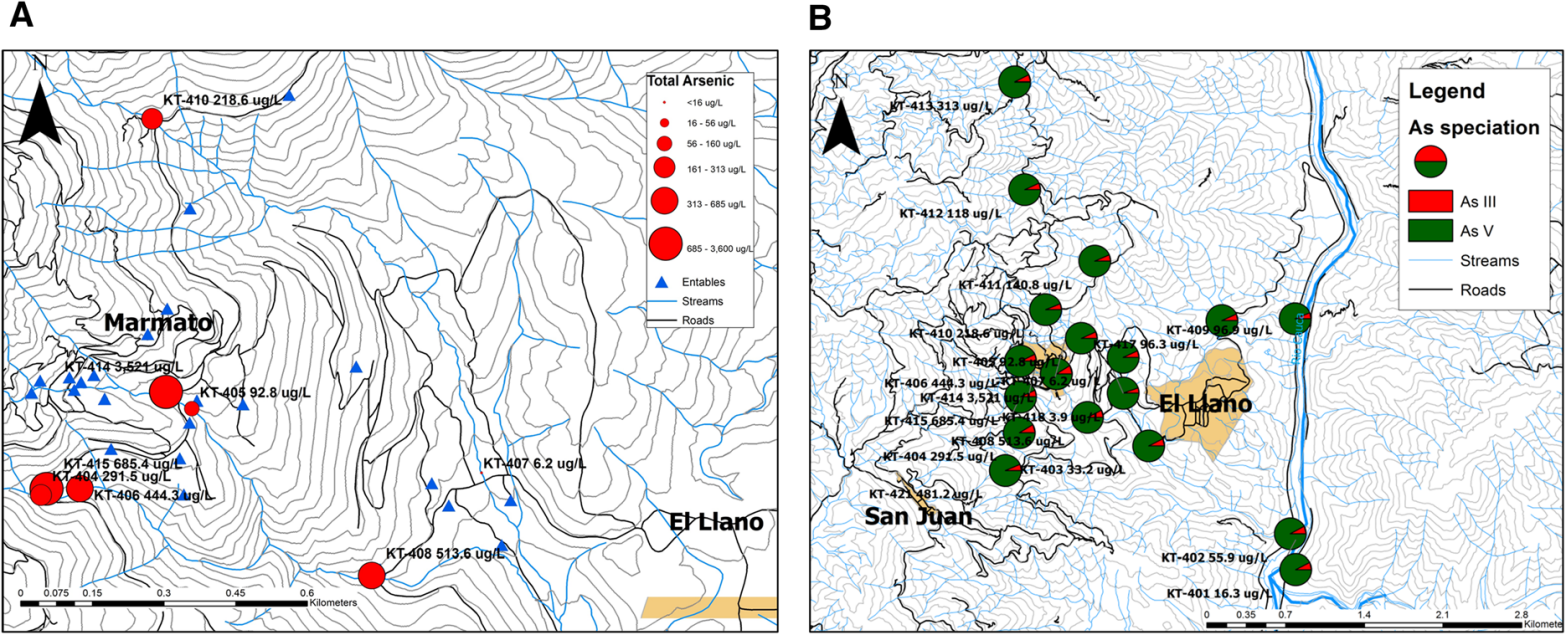
**a** Total arsenic concentration is surface water at Marmato. **b** Arsenic speciation at Marmato. The dominant species is arsenate, As(V)

Arsenic concentrations exceed the WHO’s guidance value for drinking water of 10 µg L^−1^ at all but one sampling point. Figure 8a shows total As concentrations around Marmato. Colombia has additional maximum thresholds for arsenic in irrigation water (100 µg L^−1^) and for livestock water supply of 200 µg L^−1^ (Alonso et al., 2014). Five of the water samples collected exceeded the higher threshold for livestock water.

### Arsenic speciation

Dissolved arsenic is stable under normal Eh and pH conditions in streams as either arsenite, As(III), or arsenate species, As(V) (Cullen & Reimer, 1989). It has been noted that there are no published arsenic speciation results published from sites in Colombia (Alonso et al., 2014). In this study, field separation of arsenic species in water was conducted as previously described by passing a separate aliquot through disposable chromatographic columns to separate inorganic As(III) species, with later analysis by ICP-MS. Anionic arsenate species, having a negative charge, were retained in the resin, while neutral arsenite species passed through the column unimpeded.

The results, which are shown in Table 1, indicated that 91 to 95% of the arsenic species is in the form of As(V), with little spatial variation (Fig. 8b). This is consistent with the measured pH and Eh values, which predict the dominance of the more oxidized As(V) form. It is also further evidence that there is minimal groundwater interaction with the streams, which would have an As(III) signature due to reducing conditions within the subsurface.

**Table 1:** Arsenic speciation in water samples at Marmato, Colombia, determined by AFS

Sample	Location	Total As µg L^−1^	As (III) µg L^−1^	As (V) µg L^−1^	% As (III)
KT-401	Rio Cauca	16.3	1.24	15.09	7.62
KT-402	Qda. Cascabel	55.9	4.36	51.52	7.81
KT-403	Qda. Cascabel	33.2	2.49	30.69	7.50
KT-404	Qda. Cascabel	291.5	25.3	266.2	8.67
KT-405	Canalon de la Iglesia	92.8	6.30	86.45	6.79
KT-406	Qda. Cascabel	444.3	31.9	412.4	7.18
KT-407	Qda. Pantanos	6.2	0.43	5.76	7.01
KT-408	Qda. Cascabel	513.6	39.3	474.3	7.65
KT-409	Qda. Los Indios	96.9	6.14	90.8	6.34
KT-410	Qda. Pantanos	218.6	13.5	205	6.19
KT-411	Qda. Los Indios	140.8	9.3	131.5	6.59
KT-412	Qda. Chaurquia	118.0	8.19	109.8	6.94
KT-413	Qda. San Jorge	313.0	23.3	289.7	7.44
KT-414	Canalon de la Iglesia	3,521	312.9	3,208	8.89
KT-415	Qda. Cascabel	685.4	46.7	638.6	6.82
KT-416	Rio Arquia	160.9	9.41	151.48	5.85
KT-417	Qda. Los Indios	96.3	6.57	89.69	6.82
KT-421	Qda. Aguas Claras	481.2	30.53	450.63	6.34

Qda. Cascabel has some of the highest concentrations of dissolved total arsenic and is fed by smaller tributaries, e.g., Canalon de la Iglesia, into which discharges from entables in Marmato are most severe.

### Cadmium

**Fig. 9:**
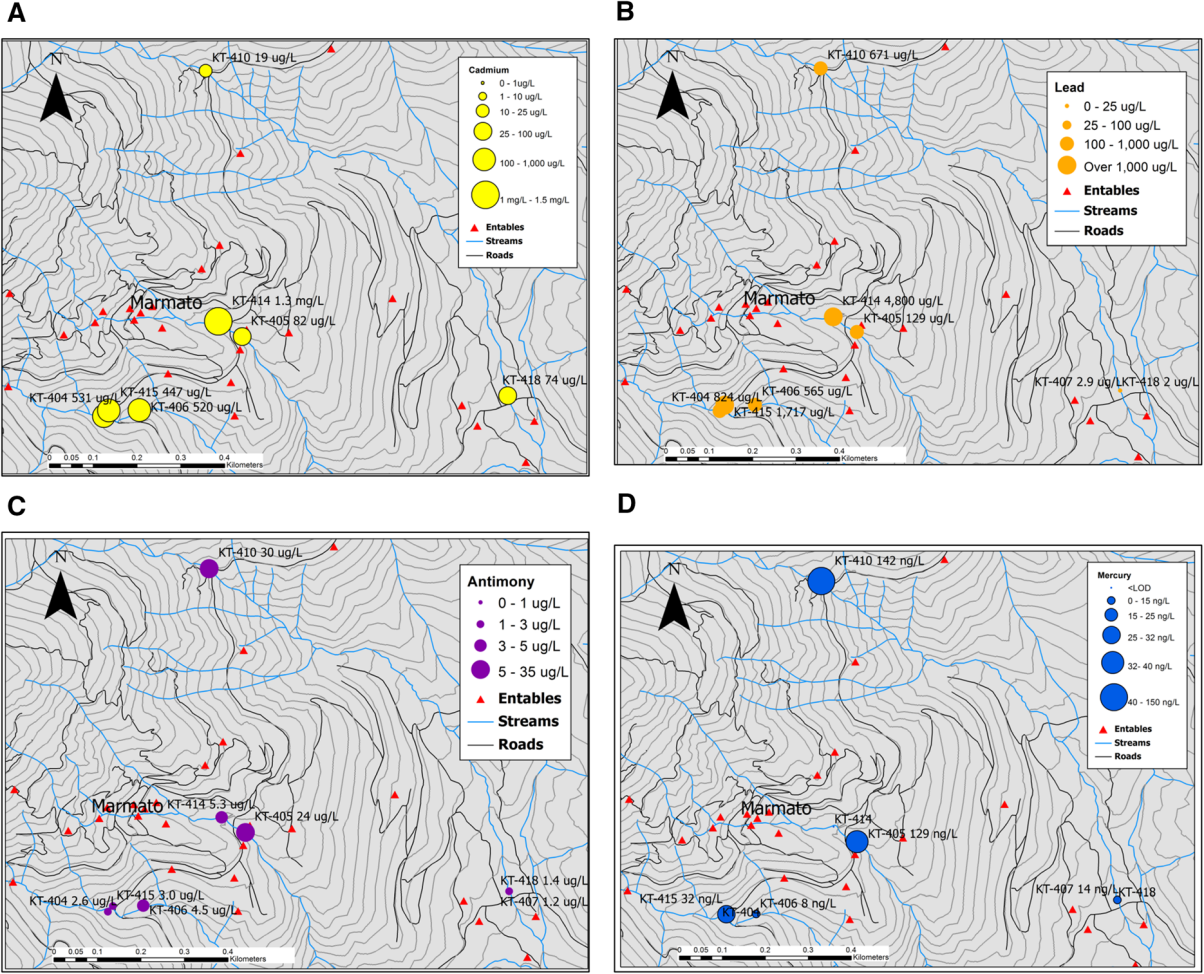
Spatial relationship between dissolved metal concentrations in surface water around Marmato to the entables (red triangles). **a** Cadmium. **b** Lead. **c** Antimony. **d** Mercury

Cadmium is an extremely toxic metal even at low concentrations, attacking the kidney and causing itai-itai disease, an osteomalacia with various grades of osteoporosis accompanied by severe renal tubular disease from chronic exposure (WHO, 2011). Total dissolved Cd in the Marmato samples ranged from 0.2 to 833 µg L^−1^. For comparison, the WHO guidance value for drinking water is 3 µg L^−1^. Cd concentrations are below 1 µg L^−1^ in Qda. Los Indios and the Rio Cauca, indicating background levels that are near normal. Cd is enriched within the deposit by a factor of 100 compared to crustal averages (Table 4). It seems likely that Cd is released from the mineral sphalerite (ZnS) which is abundant in the ore veins and is crushed with the ore; dissolved Cd and Zn concentrations show a strong correlation (*R*^2^ = 0.887). Figure 9a shows Cd aqueous concentrations in the vicinity of Marmato. Other trace metals, such as gallium and indium, were present at detectable levels which may also originate the minerals sphalerite (ZnS) and marmatite ((Fe)ZnS), which are abundant in the mineralized zones within Marmato Mountain.

### Lead

Dissolved lead (Pb) concentrations ranged from 4.4 to 4,880 µg L^−1^, compared to the WHO guidance of 10 µg L^−1^. The highest Pb concentrations corresponded to streams with the highest levels of observed suspended sediment, such as Canalon de la Iglesia (KT-415). Figure 9b shows Pb levels in stream water around Marmato. Lead in surface water probably originates from the dissolution of the mineral galena (PbS), which is concentrated during ore processing. Concentrations of lead are higher in streams that have a pH of > 5.5 and < 7.5.

### Mercury

Mercury (Hg) was detected in several samples from Qda. Cascabel (Fig. 9d), with concentrations up to 142 ng L^−1^, which is well above background levels, which are below detection limits (10 ng L^−1^). WHO has set a guideline of 6 µg L^−1^ for inorganic mercury in drinking water (WHO, 2011), but this is rarely exceeded as inorganic mercury compounds are poorly soluble. Ingestion of methyl mercury is a more potent exposure pathway; analysis of fish tissue is a more appropriate matrix for assessing mercury impacts in a watershed. Nevertheless, the concentrations of Hg at Marmato are comparable to dissolved concentrations reported from a stream traversing an abandoned mercury mine in Alaska (Torrance et al., 2012b).

**Fig. 10:**
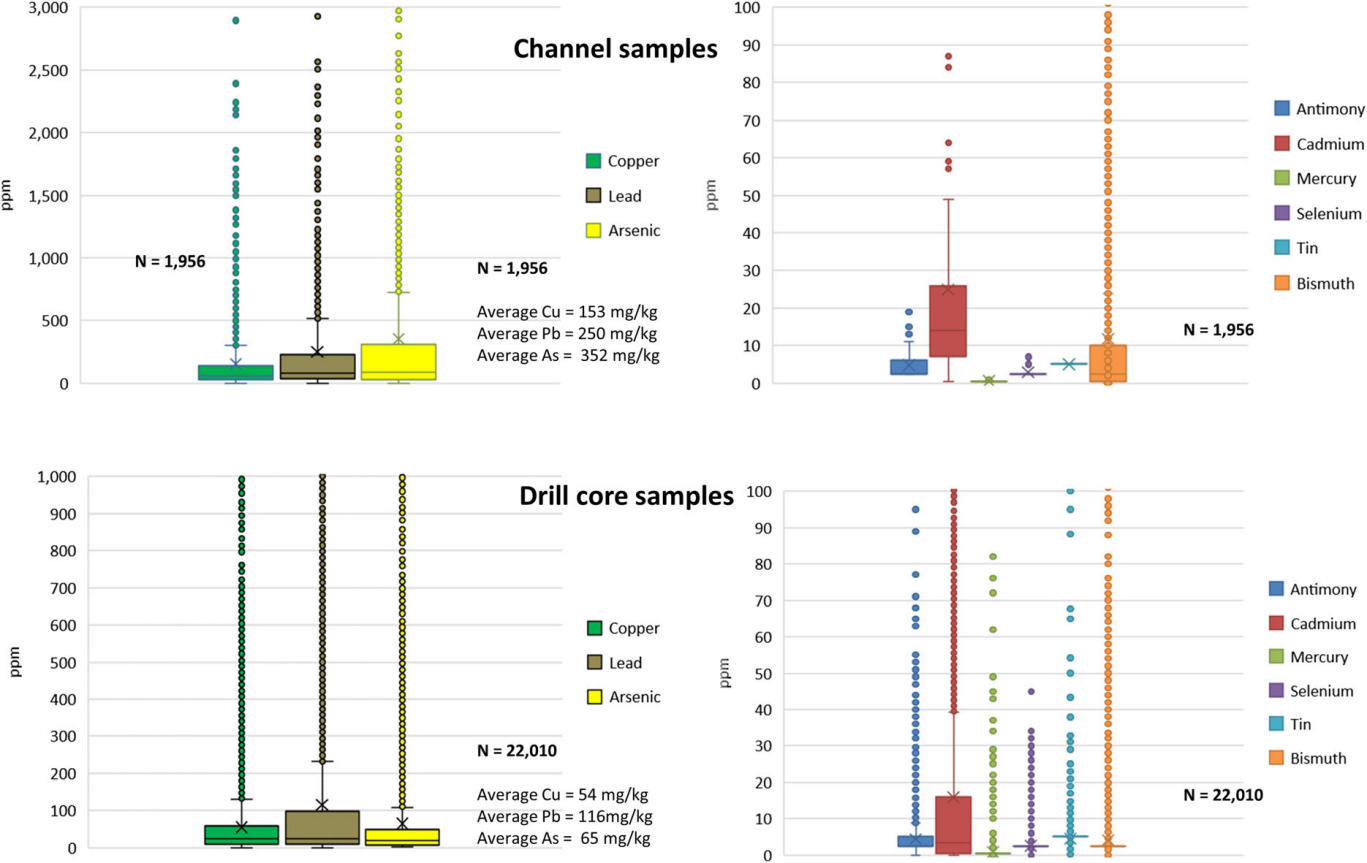
Box and whisker charts showing metal concentrations by atomic absorption (AA) and ICP analytical methods in surface channel samples (top figures) and diamond drill core (bottom figures)

Leaching studies of representative mining waste carried out to determine the acid rock drainage (ARD) potential at Marmato indicated a maximum dissolved Hg concentration of 10 ng L^−1^ in the leachate from a single sample out of 20 tested (Knight Piésold Consulting, 2012). It seems therefore unlikely that dissolved Hg in the streams around Marmato originates from naturally occurring minerals in the ore and that Hg is more likely to have been added during ore processing to enhance gold recovery. This is corroborated by the lack of enrichment of Hg in the deposit as shown by multi-element analyses of drill core samples with a mean value of 0.886 ppm and a range of < 0.005 ppm (lower limit of detection) to 373 ppm (*n* = 16,783), compared with a crustal average of 0.5 ppm (Fig. 10). Less than 10% of samples analysed had mercury concentrations above the crustal average.

The locations of the anomalous Hg sample points, shown in Fig. 9d, are not directly related to the position of the entables. This may indicate isolated mercury use by artisanal miners at some mining locations, perhaps to enhance recoveries from gold panning. No attempt was made to determine whether the Hg is present as inorganic mercury or as more toxic organo-mercury compounds.

Entables in the mining districts of the State of Antioquia of Colombia, north of Medellin, are known to make extensive use mercury to extract gold from ore through amalgamation (Hentschel et al., 2002; Prieto & Gonzalez, 1998). High levels of atmospheric mercury have been recorded in Segovia, Zaragoza, and other towns in the district (Cordy et al., 2011) where the raw ore is processed, and gold refined from doré. The United Nations Environmental Program (UNEP) estimates that artisanal gold mining accounts for the release of over 1,000 metric tons of Hg into the environment worldwide every year (Telmer & Veiga, 2009). Further, the monopolistic supply of mercury by gold buyers to the miners is viewed as an “agent of poverty” (Hilson & Pardie, 2006). An inventory of mercury in Colombia for 2011 estimated that 140 metric tons of mercury are released into the environment each year from artisanal and small-scale mining (Brooks, 2012).

Not only are non-mercury gold extraction processes more environmentally friendly; gold recovery is potentially much higher (García et al., 2015). At Marmato, although there was no visible evidence of mercury use in the entables that were visited, it is understood that mercury is frequently used in the final process to extract gold from the concentrate. As confirmation, mercury was detected in some water samples at levels up to 142 ng L^−1^.

### Antimony

Antimony (Sb) concentrations in surface water ranged from below the level of detection to a maximum of 30.2 µg L^−1^, as compared to the WHO drinking water guidance on antimony of 20 µg L^−1^ (WHO, 2011). Other regulatory agencies, such as the United States Environmental Protection Agency, have set a lower Maximum Contaminant Level (MCL) for Sb of 6 µg L^−1^. In comparison with other dissolved metals in the streams, Sb is not at especially elevated levels, although it is present in mineralized zones within the deposit. Figure 9c shows Sb concentrations in streams around Marmato.

### Other metals

Dissolved gold is present in some water samples up to 108 µg L^−1^. Their sampling location downstream of the entables suggests that these high values are losses from cyanide processing of crushed gold ore. It highlights the inefficiency of the gold recovery process in the entables and the associated loss of income to the miners. Concentrations of gallium and indium were also elevated; however, indium levels may be an artefact of ICP-MS analysis, which uses indium as an internal standard. Indium and gallium most likely occur as chemical substitutions in the mineral sphalerite, which is an abundant sulphide in the deposit.

Water samples from Qda. Cascabel showed elevated rare earth elements (REEs) including Nd, Eu, Gd, and Er. As there are no observed pegmatite veins in the complex, which are often elevated in REEs, their origin is unknown. For all samples, dissolved arsenic concentration was observed to correlate with zinc (*R*^2^ = 0.68), cadmium (*R*^2^ = 0.65), lead (*R*^2^ = 0.54), and iron (*R*^2^ = 0.43) dissolved concentrations, arsenic concentrations correlated less strongly with selenium (*R*^2^ = 0.35), cobalt (*R*^2^ = 0.34) and nickel (*R*^2^ = 0.26). There was no correlation (*R*^2^ < 0.07) of dissolved arsenic concentrations with antimony, copper, manganese, or barium. Dissolved mercury concentrations also showed no correlation with dissolved arsenic concentration, with reported concentrations of mercury above the detection limit confined to Qda. Cascabel, Qda. Pantanos, and their tributaries. Our interpretation of the spatial data is that dissolved mercury concentrations are related to clandestine mercury amalgamation within entables that discharge into these creeks.

### Whole rock analysis

Elemental analysis of drill cores through the mountain is presented as box and whisker diagrams in Fig. 10 and as Appendix, Table 4. Distributions for most potentially toxic metals, including As, Cd, Pb, and Sb, are positively skewed, reflecting the elevated concentrations of these metals within the mineralized areas and their association with sulphides such as arsenopyrite, sphalerite, galena and tennantite-tetrahedrite that occur in the low to intermediate sulphidation epithermal veins. Based on average crustal abundances, metals are enriched within the mineralized zone as follows: Cd > Au > As > Ag > Sb > Zn > Hg > Pb. Cu, Mo, Cr, and Sn are enriched within the deposit by a factor of less than 2 and have neither economic value nor environmental concerns.

## Conclusions

Surface water quality within the vicinity of Marmato is significantly impaired by many pollutants resulting from ASGM and gold ore processing. These impacts are as follows:

1. The disposal of milled ore slurry from the mine workings and entables visibly impacts the clarity of surface water due to very high suspended sediment loads. This renders surface water unsuitable either as a drinking water source or for agriculture.
2. Water exiting mine adits is acidic due to the oxidation of pyrite and sphalerite, with a minimum pH of 3.85 observed. Surface water downgradient of the entables has elevated pH (maximum pH 10.3) due to the addition of caustic sodium hydroxide pellets during cyanide treatment of crushed ore to extract gold.
3. Although cyanide was not one of the analytes in this study, its presence in surface water can be inferred by the observation of blue ferrocyanide staining downgradient of the entables.
4. Sphalerite, galena, and pyrite minerals associated with gold-bearing ore release cadmium, lead, and arsenic, respectively, into solution during ore processing via oxidation and physical processes.
5. Cadmium, lead, and arsenic are present in elevated concentrations within the mineralized zone of the Marmato deposit. The naturally occurring concentrations of these toxic metals in surface waters are exacerbated by mining and ore processing. Further, mining spoil that is dumped on the steep hillside further-enhances frequent mass wasting events, such as landslides and mud flows. These impacts limit evaluation of the natural, pre-mining metal concentrations in surface waters.
6. Lead, arsenic and cadmium are present in surface water at concentrations that greatly exceed WHO guidance (WHO, 2011) for drinking water quality. Other factors, such as sediment load and alkalinity, render water from these sources unsuitable for human consumption or for irrigation.
7. The source of elevated mercury concentrations in surface water is most likely from amalgamation of ore concentrates by artisanal miners, as Hg is not significantly elevated in the Marmato deposit. It has been estimated that 30–50% of mercury is not recovered during amalgamation in the entables (García et al., 2015).
8. Detection of dissolved gold up to 108 µg L^−1^suggests that the existing extraction methods do not recover all of the gold present in the ore. More efficient extraction technology could be introduced in Marmato that would be both environmentally friendly and more profitable for the miners.

The human health risks, both chronic and acute, related to ingestion of arsenic, cadmium and lead via drinking water are well documented and associated with mining districts (Candeias et al., 2019). Potable water for the use of Marmato residents is piped in from more distant sources unaffected by mining. In this study, a sampling location at Qda. Los Indios (KT-417) was considered as representative of background water quality, as Marmato residents use this stream as an alternative source of clean water. However, the polluted streams around Marmato discharge into the Rio Cauca, which is the main source of drinking water for several downstream communities and is further impacted by other mining and industrial activities in the region. Human health impacts from exposure to mercury from small-scale mining are linked to inhalation of mercury vapour (Gibb & O’Leary, 2014), and to the ingestion of methyl mercury in fish and other food sources (Palacios-Torres et al., 2018; Selin, 2009), rather than ingestion via drinking water. Impacts of artisanal gold mining in similar mining communities in western Colombia are well documented (Gutierrez-Mosquera et al., 2018; Marrugo-Negrete et al., 2017).

It has been estimated that ASGM accounts for around 87% of the gold produced in Colombia (Veiga & Marshall, 2019), which operates under a complex regulatory regime. The socio-technical interactions between large-scale and artisanal miners in Marmato have been discussed in depth (Holley et al., 2020). Gran Colombia Gold Corporation’s original proposal to develop a large-scale open pit mine at Marmato caused conflict in part because the excavation of the open pit would require the relocation of the town of Marmato. Further, the removal of most of the Zona Alta to create the open pit would greatly restrict ASGM in the district. Although much of this mining is illegal under Colombian law, it is the main source of employment in Marmato.

A modern gold mine operating under a robust permitting regime with appropriate environmental controls would avoid the extreme water quality impacts observed at Marmato. If it is accepted that restriction of ASGM is neither feasible nor socio-economically desirable, there are multiple obstacles to addressing water pollution arising from artisanal mining. The implementation of the Minamata Convention on Mercury in 2017, which was ratified by Colombia in 2019 (UN Environmental Programme, 2020), has formalized restrictions on mercury use but is unlikely to completely eliminate the practice and barriers still remain for the implementation of alternative extraction retort technologies (Bosse Jønsson et al., 2013; Clifford, 2014). A more realistic approach might be to encourage ore processing at a central facility (Veiga et al., 2014), instead of at the numerous small entables in the region. This would permit better environmental controls to be implemented, and the quality of the discharges to the River Cauca to be controlled and monitored.

## Appendix

See Tables

**Table 2:** Details of sampling points at Marmato, Colombia, with water parameters measured in the field

Sample #	Date	Location	Appearance	Latitude	Longitude	Elevation (m)	Alkalinity ppm	Temp °C	pH	Eh (mV)	DO ppm	Conductivity µS cm^−1^	TDS ppm
KT-401	1/12/12	Rio Cauca	Brown	5.461244015	−75.58018701	699	40	24.02	7.55	269.0	6.88	140	71
KT-402	1/12/12	Qda. Cascabel	Dark grey slurry	5.461743996	−75.58062304	688	31	22.43	7.43	256.2	7.89	832	438
sKT-403	1/12/12	Qda. Cascabel	Dark grey slurry	5.468780017	−75.59194397	931	35	23.52	6.67	239.3	7.26	1614	831
KT-404	1/12/12	Qda. Cascabel	Brown	5.472578024	−75.60228104	1,272	0	21.41	4.42	391.5	7.56	507	272
KT-405	1/12/12	Canalon de la Iglesia	Dark grey slurry	5.474212999	−75.59943899	1,279	75	20.87	10.30	76.3	7.99	2387	1295
KT-406	1/12/12	Qda. Cascabel	Cloudy brown	5.472701993	−75.60155198	1,241	5	21.54	5.09	296.2	7.58	428	229
KT-407	1/12/12	Qda. Pantanos	Dark grey slurry	5.47299603	−75.59398103	1,024	30	25.67	6.87	239.5	7.12	1537	759
KT-408	1/12/12	Qda. Cascabel	Dark grey slurry	5.471066013	−75.59605102	1,039	0	22.51	4.48	380.3	7.78	592	311
KT-409	1/12/12	Qda. Los Indios	Clear	5.478941984	−75.58023596	689	55	22.30	8.14	238.6	7.85	240	127
KT-410	1/11/12	Qda. Pantanos	Dark grey slurry	5.479673976	−75.60018799	1,311	100	21.06	9.17	184.2	6.43	1082	585
KT-411	1/11/12	Qda. Los Indios	Clear water	5.483569968	−75.59625202	1,359	5	19.43	8.20	217.6	4.87	174	97
KT-412	1/11/12	Qda. Chaurquia	Relatively clear	5.489267986	−75.60185599	1,483	10	18.63	8.03	215.8	6.43	70	40
KT-413	1/11/12	Qda San Jorge	Light brown	5.497889025	−75.60265596	1,510	17	19.09	7.85	217.3	6.46	133	75
KT-414	1/11/12	Canalon de la Iglesia	Dirty sediment rich	5.474526985	−75.59992899	1,317	0	22.91	4.76	251.1	6.03	595	310
KT-415	1/11/12	Qda. Cascabel	Dark grey slurry	5.472696042	−75.60217802	1,281	5	21.79	5.97	238.9	6.42	386	206
KT-416	1/12/12	Rio Arquia	Brown	5.51670799	−75.57840401	677	38	20.70	7.93	233.5	8.42	91	49
KT-417	1/12/12	Qda. Los Indios	Clear	5.478797983	−75.58608896	899	71	22.33	8.04	238.8	7.41	215	113
KT-420	1/12/12	Mine Adit	Clear, orange precipitation	5.472685983	−75.60201198	1,273	0	21.51	3.85	580.8	6.75	1348	722

^2^,

**Table 3:** Analysis of selected dissolved metals from Marmato, Colombia, by ICP-MS

Sample	Location	Appearance	Co µg L^−1^	Ni µg L^−1^	Cu µg L^−1^	Zn mg L^−1^	As µg L^−1^	Cd µg L^−1^	Sb µg L^−1^	Au µg L^−1^	Hg ng L^−1^	Pb µg L^−1^
**KT-401**	Rio Cauca	Brown	5.1	26.4	43.0	0.15	16.3	0.2	0.2	< LOD	< LOD	4.4
**KT-402**	Qda. Cascabel	Dark grey slurry	5.9	18.1	1,129	0.71	55.9	4.3	1.8	6.6	39	45.6
**KT-403**	Qda. Cascabel	Dark grey slurry	15.0	27.8	887.8	4.43	33.2	25.9	0.5	7.4	24	27.3
**KT-404**	Qda. Cascabel	Brown	87.1	75.5	402.0	182.59	291.5	530.9	2.6	5.3	< LOD	823.7
**KT-405**	Canalon de la Iglesia	Dark grey slurry	9.3	60.4	24.9	28.55	92.8	82.0	23.5	108.6	38	129.1
**KT-406**	Qda. Cascabel	Cloudy brown	104.0	88.3	241.9	153.69	444.3	519.6	4.5	7.3	8	564.6
**KT-407**	Qda. Pantanos	Dark grey slurry	40.6	38.8	1.5	19.76	6.2	90.2	1.2	0.2	14	2.9
KT-408	Qda. Cascabel	Dark grey slurry	215.7	219.5	650.0	210.28	513.6	832.8	2.7	13.1	< LOD	319.1
KT-409	Qda. Los Indios	Clear	< LOD	2.7	9.8	0.22	96.9	0.4	0.7	0.1	< LOD	4.4
KT-410	Qda. Pantanos	Dark grey slurry	1.5	57.3	200.8	26.12	218.6	18.8	30.2	37.4	142	671.1
KT-411	Qda. Los Indios	Clear water	4.5	16.9	26.7	2.41	140.8	8.4	1.8	0.5	< LOD	63.8
KT-412	Qda. Chaurquia	Relatively clear	0.3	9.1	25.0	1.95	118.0	8.1	0.7	0.1	< LOD	75.1
KT-413	Qda. San Jorge	Light brown	7.0	18.4	175.5	1.56	313.0	4.7	3.8	0.0	< LOD	691.1
KT-414	Canalon de la Iglesia	Dirty, sediment	261.1	280.1	1,974	317.11	3,521	1,307	5.3	41.9	< LOD	4,880
KT-415	Qda. Cascabel	Dark grey slurry	71.6	63.5	457.9	153.78	685.4	446.7	3.0	7.7	32	1,717
KT-416	Rio Arquia	Brown	< LOD	2.6	27.0	0.31	160.9	1.0	0.8	0.1	< LOD	38.9
KT-417	Qda. Los Indios	Clear	< LOD	1.7	6.3	0.26	96.3	0.4	0.7	0.1	< LOD	6.1
KT-419	Field Blank	Clear	< LOD	0.6	4.3	0.14	0.4	0.3	0.0	< LOD	< LOD	0.6
KT-421	Qda. Aguas Claras	Clear water	< LOD	2.2	5.3	0.24	481.2	0.4	1.4	< LOD	< LOD	7.2
RSD of analytes better than 10%												

3,

**Table 4:** Elemental assay of selected metals from drill core samples (*n* = 22,009)

	Au ppm	Ag ppm	Cu ppm	Mo ppm	Pb ppm	Zn ppm	As ppm	Sb ppm	Sn ppm	Hg ppm	Cd ppm	Cr ppm
*Crustal average*	*0.0031*	*0.08*	*50*	*1.2*	*14*	*79*	*1.5*	*0.2*	*2.1*	*0.067*	*0.15*	*100*
Average	0.31	2.37	54.40	2.22	115.98	909.44	64.85	4.36	4.08	0.684	15.83	162.74
Max	180	2,302	12,400	417	40,400	133,200	10,001	944	108	373	2,003	2,477
Min	0.003	0	0.25	0.025	0.25	0	0.5	0	0	0	0.01	0.5
Std. Dev	2.48	28.24	168.45	6.15	569.06	2814.78	278.09	10.10	2.89	5.331	49.18	128.65
Median	0.032	0.9	23	1	23	259	18	2.5	5	0.5	3.33	147
Enrichment	100.0	29.6	1.1	1.9	8.3	11.5	43.2	21.8	1.9	10.2	105.5	1.6

Average crustal values are shown in parenthesis (Barbalace, 2020) for comparison

Analyses were performed by Barringer Laboratories Inc, SGS del Peru S.A.C and Inspectorate America Corporation in support of the exploration program, from 1996 to 2008

^4^

## Footnotes

1 Analytical results from Canalon de la Iglesia (KT-409) were excluded from the calculated average as dissolved metal concentrations are almost an order of magnitude higher than from other sampling points.
